# HIV among people who inject drugs in India: a systematic review

**DOI:** 10.1186/s12889-022-13922-2

**Published:** 2022-08-10

**Authors:** Lucy Ngaihbanglovi Pachuau, Caterina Tannous, Mansi Vijaybhai Dhami, Kingsley Emwinyore Agho

**Affiliations:** 1 School of Health Sciences, Western Sydney University, Campbelltown Campus, Locked Bag 1797, NSW DC1797 Penrith, Australia; 2 Translational Health Research Institute (THRI), Western Sydney University, Campbelltown Campus, Penrith, NSW 2571 Australia; 3 Belmont Hospital, 16 Croudace Bay Road, Belmont, NSW 2280 Australia; 4 African Vision Research Institute (AVRI), University of KwaZulu-Natal, Westville Campus, Durban, 3629 South Africa

**Keywords:** Human immuno-deficiency virus, People who inject drugs, Co-infection, India

## Abstract

**Background:**

Little is known about the epidemiology of HIV infection among HIV positive people who inject drugs (PWID) in India. Injecting drug use has emerged as an important route of HIV transmission in India. The objective of this study was to conduct a systematic review on the risk behaviours associated with HIV infection among HIV positive PWID and assess the data reported.

**Methods:**

A systematic search of six electronic databases, Scopus, PubMed, PsycINFO, CINAHL, Embase and Ovid Medline was conducted. These databases were searched for published studies on injecting risk behaviours, sexual risk behaviours and socio-demographic factors associated with HIV infection among HIV positive PWID in India.

**Results:**

A total of 15 studies were included in the review of which 3 studies evaluated HIV/HCV coinfection among HIV positive PWID. Older age, low educational level and employment status were significantly associated with HIV infection. Sharing of syringe and needle, frequency of injection, early initiation of injecting practice, inconsistent condom use and having multiple sexual partners were all commonly associated with HIV infection among HIV positive PWID.

**Conclusion:**

Our study identified significant injecting and sexual risk behaviours among HIV positive PWID in India. There is an increasing HIV transmission among PWID in different states, more so in the northeastern states and in metropolitan cities in India. More studies need to be conducted in other regions of the country to understand the true burden of the disease. The lack of sufficient data among HIV positive female PWID does not preclude the possibility of a hidden epidemic among female PWID. The need of the hour is for the prevention of further transmission by this high-risk group through the provision of comprehensive programs, surveillance and robust continuation of harm reduction services.

**Supplementary Information:**

The online version contains supplementary material available at 10.1186/s12889-022-13922-2.

## Background

The high prevalence of Human Immuno-deficiency Virus (HIV) among many populations of injecting drug users (IDUs) represents a substantial global health challenge and injecting drug use is an increasingly significant cause of HIV transmission in most countries worldwide [1]. An estimated 11.3 million people globally inject drugs [2]. Injecting drug use is a significant public health concern and causes high morbidity and mortality owing to the risk of drug overdose and blood-borne infection mainly HIV and Hepatitis B and C and these are transmitted through the sharing of contaminated needles and syringes and risky sexual behaviours of those who have been infected [2]. Injecting drug use is estimated to account for approximately 10 percent of HIV infections worldwide and 30 percent of all HIV cases outside of Africa [3].

Injecting drug use (IDU) has emerged as an important route in HIV transmission in India. HIV was detected in India in 1986 among female sex workers and since then the prevention and transmission of HIV was focused with the commercial sex industry. Unfortunately, because of this, HIV transmission among people who inject drugs (PWID) and the drug-sex interface received little attention [4]. Current report on the overview of HIV epidemic in India shows that the adult prevalence of HIV is highest among PWID [5]. There are an estimated 200,000 PWID in India and the HIV prevalence among them is estimated to be 6.23 percent [6]. The integrated biological and behavioural surveillance (2014–2015) data reported a 9.9 percent national prevalence of HIV among PWID [7]. Surveillance data for 2008–2009 in India shows declining HIV infections among female sex workers but shows an increasing trend in HIV among injecting drug users and men who have sex with men [8]. Managing the spread of HIV from PWID to the general population as well as to other PWID is crucial. Coinfection of hepatitis C virus (HCV) in PWID are also cases that needs to be considered [9].

Injecting drug use has been the principal driver of the HIV epidemic in northeastern states of India, this could be due to its proximity to the ‘golden triangle’ of heroin production (Myanmar, Thailand and Laos) which has fueled much higher rates of injecting drug use than in other states of the country [10]. However, recent studies have shown an increase in injecting drugs in north and central Indian states, with buprenorphine and other pharmaceutical drugs as their drug of choice and it’s easy availability through pharmacies [11]. People who inject drugs (PWID) are often subjected to marginalization and stigmatization which creates social and economic barriers to access public health interventions. Despite these significant concerns there is little understood about HIV infection among PWID in India.

Harm reduction which includes needle/syringe programs and opioid substitutions is an evidence-based approach to HIV prevention and treatment for PWID and is supported by World Health Organization (WHO) and other UN agencies [12]. In India, under the National AIDS Control Program (NACP) harm reduction comes in a package of services which includes Needle Syringe Exchange Programmes (NSEP), Opioid Substitution Therapy (OST), peer-education for adopting safer behaviours, primary medical care and referral for other health care needs. This package of interventions is collectively called ‘Targeted Interventions’ (TIs) and is typically delivered by Non-Government Organizations (NGOs) working with PWID [13]. Some of these TIs have shown improvements in safe injection practices and consistent condom use with regular sexual partners but non-decline in HCV and HIV prevalence [14].

Our aim was to explore factors associated with HIV infection among PWID in India by conducting a systematic review of peer- reviewed literature reporting data on the epidemiology of HIV and the sociodemographic, injecting and sexual behaviours associated with HIV among HIV positive PWID.

## Methods

### Search strategy and data sources

The review was conducted using the 2020 Preferred Reporting Items for Systematic Reviews and Meta-analysis (PRISMA) guidelines [15]. The review was registered with the International Prospective Register of Systematic Review (PROSPERO) and the registration number is CRD42021240957. We systematically search six databases Scopus, Medline, PubMed, PsycINFO, CINAHL and Ovid Embase for studies published between January 2000 to April 2021. The year 2000 was used as a baseline in this review as this was the year the Millennium Development Goals (MGD) was introduced by the United Nations to combat different social inequalities and diseases including HIV [16, 17].

Relevant MesH words and sub-headings were used to generate articles from the six databases. The following MesH terms and keywords were used in the search:

- (HIV infections or HIV seroprevalence or Prevalence or Human Immunodeficiency Virus)

AND

- (Substance abuse, intravenous/ or Drug user*/ or people who inject drug* or injecting drug use* or intravenous drug use* or injecting drug abuse* or injecting drug*)

AND

- (Risk factor* or risk-taking or needle shar* or sexual behaviour or syringe shar* or multiple sex partner* or injecting practice* or sex work* or sexual practice* or sex partner*)

AND

- (India)

### Study selection and eligibility criteria

All articles identified in the search were exported into Endnote X9, where all duplicates were removed and screening and selection of remaining articles were done. The first author (LNP) screened all the titles of remaining publications. The next phase of screening involved reading the abstracts of studies retained from the first phase and eligible articles were retained for full text reading. In the final phase (LNP) read full text of the remaining articles and were assessed for eligibility. Studies were included in the review if they meet the following criteria: i) focused on HIV among people who inject drugs only ii) recorded coinfections with hepatitis C among HIV positive PWID iii) observational studies (qualitative studies, books, reports, policy briefs, letters or thesis were excluded) iv) published between the year 2000 and 2021 v) published in a peer-reviewed journal vi) written in English vii) focused on India only.

Two authors LNP and MVD independently assessed the quality of the studies and extracted the relevant data. All disagreements between the two reviewers were resolved through discussion and consensus on potential eligibility reached. Third and fourth reviewers KA and CT adjudicated the differences that emerged in the selection of the final studies for inclusion.

### Quality assessment

The assessment tools of the National Heart, Lung and Blood Institute of the National Institutes of Health (NIH) for quality assessment of Observational Cohort and Cross-sectional Studies and Controlled Intervention Studies was used for the quality assessment of this review [18]. There are 14 items on the checklist that evaluate the potential selection bias (external validity) and potential measurement bias (internal validity) of observational studies. Scores assigned to each reviewed study range from zero to 14 points (zero if none of the criteria were met and 14 points if all the criteria were met). The overall quality of the study was determined by the number of points awarded. Studies were rated as good (≥ 11), medium (6–10), and poor (≤ 5). A low-quality rating implied a high risk of bias in the study.

## Results

**Fig. 1:**
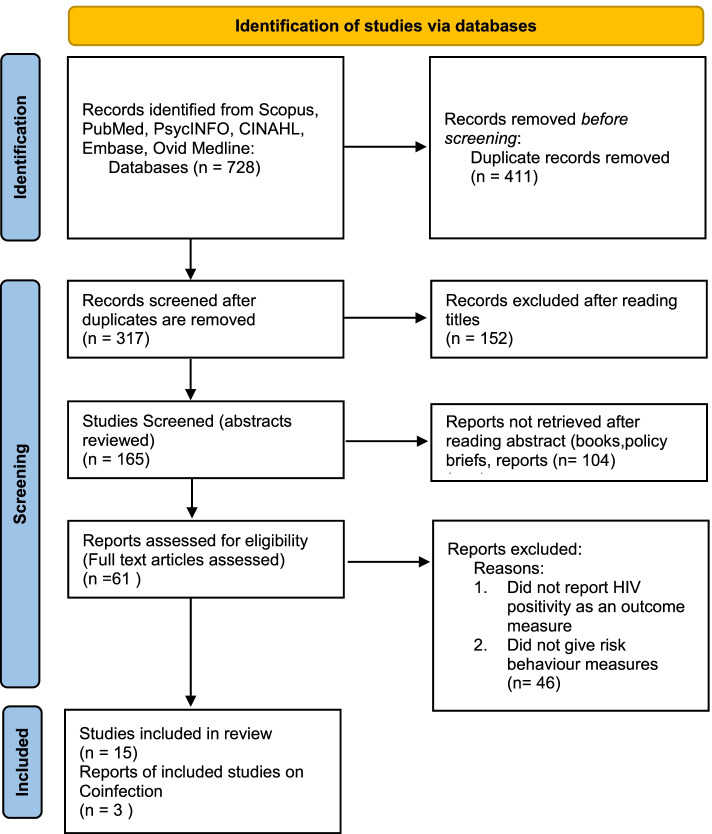
Flowchart of study selection based on PRISMA 2020 guidelines

In this systematic review a total of 728 non-duplicate records from six databases were screened. After review of titles and abstracts 67 articles were retrieved for full text review. A total of 15 articles met the inclusion criteria for this review. The review process is presented graphically in Fig. 1.

### Characteristics of the study

Tables 1 and 2 summarizes the characteristics of the included 15 studies. Of the studies conducted, 15 studies recorded the injecting risk factors among HIV positive PWID, 12 recorded the sexual risk factors and 14 studies recorded the sociodemographic risk factors of HIV positive PWID. Only 3 studies recorded the HIV/HCV coinfections and their risk factors among PWID. Sample sizes ranged from 75 to 19,902 PWID. Out of 15 studies, 6 studies were done in northeastern states of India, 4 studies were conducted at the national level and 5 studies were conducted in different states. The quality of the included studies in this review demonstrated that all 15 studies were of medium quality. The details of the specific scores are given in Supplementary Table S1.

**Table 1:** Sociodemographic, injecting and sexual risk factors associated with HIV positivity among PWID

**Author/Year**	**Geographical Region**	**Sample/Age**	**Sampling Strategy**	**Study design**	**Population characteristics**	**Number of HIV + PWID**	**Sociodemographic factors**	**Injecting risk factors**	**Sexual risk factors**	**Study Strengths**	**Study Limitations**	**Quality Assessment score**
Ganesh et al. (2020) [19]	Manipur	*n* = 1594 Aged 15 years and above	Two stage cluster sampling	Community-based, bio-behavioural surveillance	Men who inject drugs in the past 3 months of data collection	Male- 193	Labourers/manual workers, older age > 39 years, did not go to school, living alone or with friends	Injecting at their own house, sharing of needle/syringe, repeated use of needle/syringe, drawing up from same container, longer duration of injecting practices	Having multiple sexual partners	The study gave evidence on important factors associated with HIV transmission among HIV positive PWID	The study did not include female PWID	9
Kumar et al. (2018) [20]	India	*n* = 19,902, Aged 15 years and above	Conventional cluster sampling	Probability- based, cross-sectional study	Men who inject drugs in the past 3 months of data collection	Male- 1631	Older age (> 25 years), marital status (never married), Occupation (non labourers)	< 24 years of age at initiation of injecting drug use, duration of drug use (five years and above), frequency of injecting (twice/day), type of drugs (buprenorphine, heroin), injected in groups during last injection	Inconsistent condom use, reported STI symptom	The study could be generalizable to the India population due to large sample size used across 29 states in India.-To determine HIV status two test protocols were used -Information were collected by trained personnel who used standardized questionnaire to ensure consistency across all Indian States and territories	Due to the nature of the study there could have been measurement bias on leading to an overestimation or underestimation of factors -No female participants	7
Cepeda et al. (2017) [21]	15 cities in India	*n* = 14,373 Aged 18 years above	Respondent Driven Sampling (RDS)	Cross-sectional study	People who inject drugs	2915	Not given	Passing a used needle/syringe to more than 3 individuals in the past 30 days	-	The large sample size and low loss to follow up rate confers greater statistical power	Sociometric data were not obtained and because of this homophily was low (between -0.2 and 0.2) for most sites -Generalisability was limited because random sample of underlying population could not be obtained -sexual risk factors were not obtained	8
McFall et al. (2017) [22]	7 cities in Northeast India	*n *= 796 Aged 18 years above	RDS	Cross-sectional study	Injecting in the previous 2 years of data collection	Female- 368	Older age, widowed, having children, having attended secondary school	Injection of buprenorphine, longer duration of injecting use, less frequency of injection	Higher number of sexual partners	-All female participants as there are few studies that look at female PWID	-Establishment of temporality or causality is not possible due to the cross-sectional nature of the study -Sample or estimates are not representative of the underlying population	
Lucas et al. (2015) [23]	India- 15 Indian cities	*n* = 14,481 Aged 18 years and above	RDS	Cross-sectional study	Reported injecting drug use in the prior 2 years of data collection	2905	Female gender, marital status (currently married or living with a partner), age	Initiation of early injection, type of drugs injected (buprenorphine and heroin), sharing of needle/syringe	Number of lifetime sex partner, exchange of sex for money or goods, being female	RDS was used- a strategy that is suited for ‘hidden’ populations and permits weighing to produce unbiased estimates of factors of interest in the target population -Use of state-of-the-art methods to characterize recent HIV infection across sites permitting HIV incidence estimates	Sites and samples were not selected randomly and so this data cannot be considered a nationally representative. Detailed network-level risk data were not collected	8
Armstrong et al. (2014) [14]	Manipur, India	*n* = 821 PWID Aged 18 years above	RDS	Cross-sectional study	Long-term heroin injectors	M- 252	Older age(> 25 years), currently married	Sharing of needles, longer duration of injection practice, higher frequency of injection	Did not use condoms at last sex with casual or regular partners, irregular condom use	Adequate sample size. The study gave evidence on important factors associated with HIV transmission among HIV positive PWID	-Female PWID were not included -Low uptake of HIV testing. However, this data was collected in 2009 and anecdotal evidence suggests that uptake of HIV testing is likely to be higher now	7
Mehta et al. (2014) [24]	14 sites across India	*n* = 801 Aged 18 years and above	Simple random sampling from 14 locations in India by NGO that worked with PWID	Cross-sectional study	HIV positive PWID in the prior 2 years of data collection	Male- 689 Female- 112	Older age(> 30 years), gender (male), married, educational level, unemployment, low monthly wages	Injecting heroin and buprenorphine, needle and syringe sharing, daily injection practices	Men having sex with men, sex with a casual partner, any sexual intercourse	Pre-tested, pre-validated questionnaire was used -Data were collected by trained personnel	Small sample size in each site -Detailed information on sexual and injection related risk behaviour were not collected -Low female representation as most female PWID are confined to the Northeastern region	7
Panda et al. (2014) [25]	Punjab	*n* = 1155 Aged 18 years and above	Sample collected from Integrated Counselling and Testing centre (ICTC)	Community- based cohort study	injected drugs within the last 3 months of data collection	M- 338	Older age, gender (male), married, educational level, unemployment, low monthly wages	Length of time of injection, irregular supply of syringes and needles, sharing of syringe and needle	Having genital disease symptom within the last year	Wide community based study	Inability to recruit those who are no in contact with Targeted intervention services which may present a biased picture	8
Sarna et al. (2013) [26]	Delhi and neighbouring states	*n* = 3792 Aged 18 years and above	Samples were recruited through Peer referral, targeted outreach and walk-in clients	Longitudinal cohort study	Injecting drugs in the prior 3 months of data collection	795	Educational status (illiterate and class 1–6), never married, religion (Hindu), accommodation (living in streets or public places), employment status (daily wager), monthly income (1501–5000 INR)	Injecting drugs for longer periods, frequency of injections per day, sharing of needle/syringe, sharing of common container to draw drugs, split/back/front loading	Sexual intercourse in last 3 months, self-reported unsafe sex with regular partners and non-regular/paid female partners	Multiple strategies were used to recruit large number of PWID -Post-test counselling provided by trained nurses to all HIV positive participants -Interview conducted by trained research interviewers	Self-reported risk behaviours which maybe subject to social desirability bias	9
Chakrapani et al. (2011) [27]	Manipur	*n* = 75 Aged 18 years above	Convenience sample recruitment	Cross-sectional study	People who inject drugs in the past 3 months of data collection	Male- 50 Female- 25	Older age, unemployment among men, low monthly income, low educational level among women (did not complete high school), sex work as an occupation for women	Injection of heroin and methamphetamine, sharing of needles/syringe in past 30 days, type of drug used in past 3 months	Exchange of sex for drugs and money in the past 30 days	Pre-tested, pre- validated questionnaire was used	The use of convenience sample for HIV positive PWID in the survey -Small participants, a small number of indepth interviews and key informant interviews represents limitation in that saturation cannot be ensured	7
Solomon et al. (2008) [28]	Chennai	*n* = 912, Aged 18 years and above	Convenience sample recruitment	Longitudinal cohort study	Injected drugs at least once in the prior 6 months of data collection	Male- 217	Older age, ethnicity, being married, low educational level (no education or primary level) employment	Heroin injection, higher frequency of injection, sharing of injecting equipment, larger needle sharing network, injecting at dealers’ place	Less sexual activity	All participants received pre and post test counselling -Standardized questionnaire was administered by trained interviewers	-Inability to ascertain temporality of recent risk behaviours and prevalent HIV infection given the cross-sectional design	8
Panda et al (2005) [29]	Chennai, India	*n* = 226 Aged 18 years and above	Mapping exercise for drug users was done using snowballing technique	Cross-sectional study	Injecting drugs in the previous 6 months of data collection	68	Older age, low educational status, geographical location	Early initiation of injecting drug use, having a tattoo, borrowing and lending injection equipment	Sexual debut with a commercial sex worker, inconsistent condom use	Pre-tested, pre-validated questionnaire was used	The temporality could not be established due to the cross-sectional nature of the study -Due to the small sample size the study findings are not representative of the national population of India -Detailed statistical analysis for determinants of HIV infection in women could not be determined	8

**Table 2:** Risk behaviours associated with HIV/HCV Co-infection among PWID

Author/Year	Geographical location	No. of participants/Age	Study Design	Sampling strategy	Population Characteristics	HIV /HCV Coinfection	Sociodemographic factors	Injecting risk factors	Sexual risk factors	Study Strengths	Study Limitations	Quality Assessment Score
Ray Saraswati et al. (2015) [30]	Delhi	*n* = 3792 Aged 18 years and above	Longitudinal cohort study	Mapping exercise of hot spot area was done and participants were recruited through peer-referral and targeted outreach by outreach workers	Injecting in the past 3 months of data collection	Male- 449	Older age, illiterate, never married, Hindu religion, living at home with family or either living on the street, geographical location	Longer duration of injection (2-5yrs), a greater number of days injected in the past month (21–30 days), sharing needles/syringe, sharing of injecting equipment, using syringe filled by someone else	Not sexually active in the last 3 months	Large sample size which allowed for examining sociodemographic, injecting and sexual characteristics associated with strong statistical power and analysis and minimal recall bias	-Just two-thirds of participants returned for follow up -Low female participants hence they were removed from statistical analysis	8
Kermode et al. (2014)	Manipur	*n* = 821 Aged 18 years and above	Cross-sectional study	Respondent driven sampling	Injecting at least once in the past 6 months of data collection	Male- 241	Older age ≥ 30 yrs, illiterate, widowed, divorced or separated, being employed	Earlier age of first injection, longer duration of injecting, injecting at least once daily, sharing of injecting equipment, sharing of needle/syringe	-	RDS was used to recruit study participants	-Not possible to infer causation for outcome variables due to the nature of the study design - Social acceptability bias may have contributed to an under-estimate in the prevalence of unsafe injecting behaviour	10
Mahanta et al. (2008) [9]	Nagaland and Mizoram	*n* = 398 Aged 15 years and over	Cross-sectional study	PWID who attended drop-in centers within a given time period were randomly recruited for the study	Injecting within past 6 months of data collection	Male- 34	Older age ≥ 25 yrs, male gender, married	Use of heroin, longer duration of injecting, sharing injection containers	-	Pre-tested, pre-validated structured questionnaire was used	-Due to the random recruitment strategy the study findings are not representative of the PWID population of Nagaland and Mizoram -Temporality could not be established due to the cross-sectional nature of the study	

### Prevalence and 95% confidence intervals (CI) of HIV among PWID and its related behaviours in India

Supplementary table S2 shows the prevalence and 95% CI of HIV and its related behaviours among PWID. The HIV prevalence ranged between 9.9% from the integrated biological and behavioural surveillance data to 52.9% in studies done in northeast India. Sharing of needle/syringe was the most common risky injecting behaviour among PWID and ranged from 7.8% (95%CI 1.2- 14.4) and 57.1% (95% CI 52.6–61.6). Having multiple sex partner was the most common risky sexual behaviour, the prevalence was between 6.9% and 48.6% and both of these studies were done in the northeastern states.

### Sociodemographic factors associated with HIV positive PWID

Sociodemographic factors evaluated in this review are summarized in Table 1. Many studies restricted the recruitment to PWID aged 18 or over. The review showed that older age (> 25 years) [19, 20, 22, 24, 25, 27–29, 31], low educational level [19, 22, 24–29], manual workers/daily wagers [19, 24, 26, 27], being married [23, 24, 28, 31], living in streets or public places [26] were associated with HIV positivity among HIV positive PWID. In contrast, some studies [20, 26] reported that PWID who were never married and widowed [22] were also associated with HIV infection among HIV positive PWID.

### Injecting risk behaviour associated with HIV infection among HIV positive PWID

This review found that sharing of syringes and needles [19, 21, 23–29, 31] were the most common injecting risk behaviour among HIV positive PWID. Heroin and buprenorphine were the drug of choice among this population [20, 22–24, 27, 28]. Chakrapani et al. [27] also found methamphetamine to be common among HIV positive PWID. Early initiation of injecting practice [20, 23, 29], longer duration of injecting drug use [19, 20, 22, 25, 26] and higher frequency of injecting [20, 26] were risk behaviours associated with HIV infection among HIV positive PWID. Additionally, injecting at their own home [19], injecting at a dealer’s place [28] were associated with HIV infection among this population. Interestingly, PWID who also had a tattoo were also associated with HIV infection [29]. Table 1 summarizes the injecting risk behaviour associated with HIV infection among PWID.

### Sexual risk behaviour associated with HIV infection among HIV positive PWID

The reviewed studies demonstrated that sexual risk behaviour associated with HIV infection among HIV positive PWID included inconsistent condom use [20, 29, 31], having multiple sexual partners [19, 22, 23, 26], exchange of sex for drugs and money [23, 27], men having sex with men [24], having sexually transmitted infection (STI) symptoms [20, 25] and sexual debut with commercial sex worker [29]. In contrast, Solomon et al. [28] reported that HIV positive PWID had less sexual activity. Table 1 summarizes the sexual risk behaviour associated with HIV infection among HIV positive PWID.

### HIV/HCV coinfection among HIV positive PWID

Three studies were found in this review that reported HIV/HCV coinfection among HIV positive PWID ( see Table 2). The reviewed studies demonstrated that older age (≥ 25) [32–34], being illiterate [32, 33], never married, widowed, divorced or separated [32, 33] and male [34] were the sociodemographic factors associated with HIV/HCV coinfection among HIV positive PWID. The papers reviewed for the study also found that longer duration of injection, sharing of syringe and needle were associated with HIV/HCV coinfection.

## Discussion

Most of the studies included in this review had generated survey-based estimates of HIV prevalence among PWID. Among the papers reviewed, HIV prevalence among PWID is highest in the northeastern states of India particularly the state of Manipur. This could be attributed to its geographical location, sharing border with Myanmar, resource limitation and socio-political problem which have all contributed strongly on spreading HIV and failure of preventive program of HIV/AIDS [35]. India-Myanmar border has a unique arrangement called the Freedom Movement Regime (FMR). The FMR permits the tribes residing along the border to travel 16 KM across the boundary without Visa restrictions. Literature suggests that this passageway has been misused to smuggle in drugs and other contraband which resulted in high rates of injecting drug use in the northeastern states of India [36].

Most of the studies in this review were limited to male participants only thereby, limiting data among female HIV positive PWID. From the limited data available for HIV positive female PWID it appeared that a low educational level [27], having a higher number of sexual partners [22] and sharing of syringes and needles were strongly associated with HIV infection. This is similar to the studies done in San Francisco [37] and Cambodia [38]. Females who inject drugs are often threatened or intimidated with physical and sexual violence to engage in syringe/needle sharing and high risk sexual behaviours [39, 40].

Among the manuscript reviewed the most common sociodemographic factors associated with HIV infection among PWID were older age, low educational level, and employment status (manual labourers and daily wagers). The associations between low educational level, employment status and HIV infection in this review is consistent with studies conducted in Malaysia [41] and Iran [42]. This suggests that PWIDs have difficulties in obtaining a regular job and this may be due to their low educational level and injecting lifestyle [43]. A study in Italy [44] also found HIV infection among drug users was correlated with older age and longer period of drug use suggesting that older drug users who have engaged in drug use for a longer period have subsequently been exposed longer to risk factors for infection. However, they found no correlation with level of education.

Our review showed needle/syringe sharing, frequent injecting, early initiation of injection practice and injecting of heroin and buprenorphine as proximal factors associated with HIV infection. We found sharing of syringes and needles to be the most common injecting risk behaviour among HIV positive PWID. This finding is in line with other studies that have focused on HIV among PWID [45–48]. Our review showed that PWID in India are mostly low wage earners with limited financial resources to purchase injecting equipment contributing to an increased likelihood of reuse and sharing of contaminated injecting equipment [49]. This is one of an array of reasons explaining the risky injection behaviour among PWID despite India’s efforts in promoting harm reduction intervention which includes needle and syringe exchange program (NSEP). According to several studies around the world, the main barrier for PWID in accessing NSEP is policing and the criminalization of drug users and because of this injecting drug users would rather share injection equipment and avoid harm reduction programmes due to the fear of being arrested [50–54]. In India, consumption of drugs is illegal and results in jail term of up to six months or one year and/or a fine depending on the substance consumed [55]. Decriminalization of drug use will allow injecting drug users to access harm reduction programmes, thereby decreasing HIV infection through the use of un-used and sterile needles and syringes. Portugal is an example of successful drug reform efforts, where decriminalizing drug use in 2001 led to a significant reduction in HIV infections [56].

Three papers in our review showed HIV/HCV coinfection and risk behaviours among coinfected PWID. Of the three papers, two studies were conducted in the northeastern states of India which highlights the need to further study HIV/HCV coinfection among PWID in this region. Understanding the true burden of disease at a community level as well as prevalent risk behaviours are critical for designing effective prevention interventions to curtail the spread of HIV and other infectious diseases among PWID as well as from their sexual partners and the general population [57]. The injecting and sexual risk behaviour of coinfected PWID in this review are similar to those reported with HIV infection alone. This finding is consistent with other studies which have found high-risk injection practice such as injection with a syringe used by another PWID to be the major mode of transmission of HCV [30, 58–61]

### Policy and service provision implications of the study findings

Papers reviewed for this study found that blood-borne infections such as HIV, Hepatitis C and Hepatitis B among PWID is spread primarily through injecting risk behaviour related to sharing of needles and syringes as well as through sexual risk behaviours such as unprotected sex and exchange of sex for drugs and money. India, through its National AIDS Control Program (NACP) adopted a comprehensive package of biomedical behavioural interventions as the optimal HIV prevention strategy. This comprehensive package is recommended by World Health Organization (WHO), United Nations Office on Drugs and Crime (UNODC), the joint United Nations Programme on HIV/AIDS (UNAIDS). The package consists of 9 components which include a needle syringe exchange programme (NSEP), Opioid Substitution Therapy (OST) and Targeted Information, Education and Communication (IEC).

The NACP-IV ‘Targeted Intervention’, a package that caters particularly for high-risk groups including PWID includes the delivery of services comprising of needle syringe exchange program (NSEP), opioid substitution therapy (OST), peer education for adopting safer behaviours, primary medical care, condom distribution and referral for other health-care needs [62]. These services are typically delivered by NGOs working with PWID. However, despite these efforts the service coverage remains insufficient and are available to only a small proportion of PWIDs in India particularly due to stigma and discrimination [63]. The effectiveness of harm reduction particularly NSEP for preventing the spread of HIV among PWID has well been established and widely published [64, 65].

WHO, UNAIDS and UN office on drugs and crime has recommended mobile needle and syringe programmes as an alternative or complimentary delivery method of needle and syringe programs [66]. Mobile syringe and needle programs attract people who engage in high-risk and high-intensity injection behaviours; they reduce barriers such as stigma and exposure to local policing [67]. These mobile programs are not just limited to providing clean injecting equipment but can offer specialized interventions such as primary care, HIV treatment, education and case management [68]. Focused attention and support of the limited provision of mobile needle and syringe programs in India may increase accessibility among PWID in acquiring HIV prevention services and curtail viral transmission of HIV among PWID and their sexual and injecting partners.

However, the prevention of HIV among PWID cannot be achieved through one program or service alone but requires comprehensive package of interventions geared towards specific needs of PWID. The national and local government needs to continue robust engagement and support of PWID and continue working with other community organizations supporting PWID. This will strengthen linkage to services and increase the probability of retention in services [63].

Pre-exposure Prophylaxis (PrEP) has been recommended by WHO to be included in the HIV prevention package for PWID [69]. The Bangkok Tenofir Study [70] found daily oral Tenofir reduced the risk of HIV infection in PWID and considered PrEP with Tenofir for use as part of the HIV prevention package for PWID. In India, the use of PrEP has been available in the private sector since 2016, however, PrEP has not been rolled out as part of a public sector National AIDS Control Program. However, a new national policy is underway to roll out PrEP as part of HIV combination prevention [69]. Belludi et al. [69] highlight the need for key population-focused education and campaigns about PrEP and self-assessment of risk to link high-risk groups in PrEP programs. Future studies need to focus on the method of delivery and the effectiveness of PrEP in reducing the transmission of HIV among PWID in India.

### Strengths and limitations

The strength of our study lies in the comprehensive and exhaustive search through extensive databases and having two independent reviewers undertake the study selection, examined the studies to be included in the review based on the inclusion and exclusion criteria through discussion and consensus as well as quality assessment. However, there are also limitations to our study. One limitation of our study is that there were virtually no HIV data among HIV positive PWID in the majority of the states in India which limited the assessment of the status of the epidemic. Secondly, data on female HIV positive PWID were limited which may result in gender bias. Thirdly, studies on HIV/HCV coinfection among HIV positive PWID was limited which made it difficult to draw conclusion into the associated factors of coinfection. Fourthly, most of the included studies were cross-sectional which may have resulted in recall bias in the findings due to the nature of data collectionAnother limitation is that there is no quality rating for the choice of behaviours selected for the presentation of the results. Lastly, we acknowledge that there are some studies that are done outside of India, however these studies are beyond our study inclusion criteria.

## Conclusions

The papers reviewed for this study found significant injecting and sexual risk behaviours among HIV positive PWID in India. There is evidence for HIV epidemic among PWID in different states, more so in the northeastern states and in metropolitan cities in India. More studies need to be conducted in other regions of the country to understand the true burden of the disease. The lack of sufficient data among HIV positive female PWID does not preclude the possibility of a hidden epidemic among female PWID. The need of the hour is for the prevention of further transmission by this high-risk group through the provision of comprehensive programs, surveillance and robust continuation of harm reduction services.

## Supplementary Information

**Additional file 1.**

**Additional file 2.**

## Data Availability

The datasets used and/or analysed during the current study available from the corresponding author on reasonable request.

## Abbreviations

HIV: Human Immuno-deficiency Virus
HCV: Hepatitis C Virus
IDU: Injecting Drug Use
PWID: People who inject drugs
MGD: Millenium Development Goals
NACP: National AIDS Control Program
NSEP: Needle Syringe Exchange Program
OST: Opiod Substitution Therapy
UNAIDS: United Nations Programme on HIV/AIDS
WHO: World Health Organisation
