# Supplementary material for: HIV among people who inject drugs in India: a systematic review
Source: BMC Public Health. 2022 Aug 10;22:1529. doi: 10.1186/s12889-022-13922-2 (PMC9367073; doi:10.1186/s12889-022-13922-2)
Supplement: Supplementary file 1 — Additional file 1. [file 12889_2022_13922_MOESM1_ESM.docx]

Supplementary Table S1: Quality Assessment Score

| Author, Year | Was the research question or objective in this paper clearly stated? | Was the study population clearly specified and defined? | Was the participation rate of eligible persons at least 50%? | Were all the subjects selected or recruited from the same or similar populations (including the same time period)? Were inclusion and exclusion criteria for being in the study prespecified and applied uniformly to all participants? | Was a sample size justification, power description or variance and effect estimates provided? | For the analyses in this paper, were the exposure(s)  of interest measured prior to the outcome(s) being measured? | Were the exposure measures (independent variables) clearly defined, valid, reliable, and implemented consistently across all study participants? | Was the timeframe sufficient so that one could reasonably expect to see an association between exposure and outcome if it existed? | For exposures that can vary in amount or level, did the study examine different levels of the exposure as related to the outcome, (e.g, categories of exposure, or exposure measured as continuous variable)? | Were the exposure measures (dependent variables) clearly defined, valid, reliable and implemented consistently across all study participants? | Was the exposure(s) assessed more than once over time? X | Were the outcome assessors blinded to the exposure status of participants? | Was less to follow up after baseline 20% or less? | Were key potential confounding variables measures and adjusted statistically for their impact on the relationship between exposure(s) and outcome(s)? | Quality Assessment Score |
| --- | --- | --- | --- | --- | --- | --- | --- | --- | --- | --- | --- | --- | --- | --- | --- |
| Ganesh et al (2020) | 1 | 1 | 1 | 1 | 1 | 1 | 1 | N/A | N/A | 1 | 0 | N/A | 0 | 1 | 9 |
| Kumar et al (2018) | 1 | 1 | 1 | 1 | 0 | 1 | 1 | N/A | N/A | 1 | 0 | N/A | 0 | 0 | 7 |
| Cepeda et al (2017) | 1 | 1 | 1 | 1 | 0 | 1 | 1 | N/A | N/A | 1 | 0 | N/A | 0 | 1 | 8 |
| McFall et al (2017) | 1 | 1 | 1 | 1 | 0 | 1 | 1 | N/A | N/A | 1 | 0 | N/A | 0 | 1 | 8 |
| Lucas et al (2015) | 1 | 1 | 1 | 1 | 0 | 1 | 1 | N/A | N/A | 1 | 0 | N/A | 0 | 1 | 8 |
| Ray Saraswati et al (2015) | 1 | 1 | 1 | 1 | 0 | 1 | 1 | N/A | N/A | 1 | 1 | N/A | 0 | 0 | 8 |
| Armstrong et al (2014) | 1 | 1 | 1 | 1 | 0 | 1 | 1 | N/A | N/A | 1 | 0 | N/A | 0 | 0 | 7 |
| Mehta et al (2014) | 1 | 1 | 1 | 1 | 0 | 1 | 1 | N/A | N/A | 1 | 0 | N/A | 0 | 0 | 7 |
| Kermode et al (2014) | 1 | 1 | 1 | 1 | 1 | 1 | 1 | N/A | N/A | 1 | 0 | N/A | 0 | 1 | 9 |
| Panda et al (2014) | 1 | 1 | 1 | 1 | 0 | 1 | 1 | N/A | N/A | 1 | 0 | N/A | 0 | 1 | 8 |
| Sarna et al (2013) | 1 | 1 | 1 | 1 | 1 | 1 | 1 | N/A | N/A | 1 | 0 | N/A | 0 | 1 | 9 |
| Charapani et al (2011) | 1 | 1 | 1 | 1 | 0 | 1 | 1 | N/A | N/A | 1 | 0 | N/A | 0 | 0 | 7 |
| Mahanta et al (2008) | 1 | 1 | 1 | 1 | 0 | 1 | 1 | N/A | N/A | 1 | 0 | N/A | 0 | 0 | 7 |
| Solomon et al (2008) | 1 | 1 | 1 | 1 | 0 | 1 | 1 | N/A | N/A | 1 | 0 | N/A | 0 | 1 | 8 |
| Panda et al (2005) | 1 | 1 | 1 | 1 | 0 | 1 | 1 | N/A | N/A | 1 | 0 | N/A | 0 | 1 | 8 |
