# Supplementary material for: HIV among people who inject drugs in India: a systematic review
Source: BMC Public Health. 2022 Aug 10;22:1529. doi: 10.1186/s12889-022-13922-2 (PMC9367073; doi:10.1186/s12889-022-13922-2)

Supplementary table S2: Prevalence and 95% confidence intervals (CI) of HIV among PWID and its related behaviours in India.

| **HIV positive among PWID** | | | |
| --- | --- | --- | --- |
| Author(Year) | Prevalence | 95%CI | |
| Ganesh et al (2020) | 12.0 | 7.4 | 16.6 |
| Kumar et al (2018) | 9.9 | 8.5 | 11.3 |
| Cepeda et al (2017) | 20.1 | 18.6 | 21.6 |
| McFall et all (2017) | 52.9 | 47.8 | 58.0 |
| Lucas et al (2015) | 18.1 | 16.7 | 19.5 |
| Armstrong et al (2014) | 30.7 | 25.0 | 36.4 |
| Panda et al (2014) | 29.0 | 24.2 | 33.8 |
| Sarna et al (2013) | 20.9 | 18.1 | 23.7 |
| Solomon et al (2008) | 29.8 | 23.7 | 35.9 |
| Panda et al (2005) | 30.0 | 19.1 | 40.9 |
| **Sharing of needle/syringe/unsafe injection practice** | | | |
| Ganesh et al (2020) | 13.3 | 8.7 | 17.8 |
| Kumar et al (2018) | 26.7 | 25.5 | 27.9 |
| McFall et all (2017) | 48.1 | 43.1 | 53.1 |
| Armstrong et al (2014) | 7.8 | 1.2 | 14.4 |
| Panda et al (2014) | 39.0 | 34.5 | 43.5 |
| Sarna et al (2013) | 40.1 | 37.7 | 42.5 |
| Solomon et al (2008) | 29.3 | 23.8 | 34.8 |
| Mehta et al (2013) | 57.1 | 52.6 | 61.6 |
| Chakrapani et al (2011) | 33.0 | 14.5 | 51.5 |
| **Longer duration of drug use (> 5years)** | | | |
| Kumar et al (2018) | 73.8 | 73.1 | 74.5 |
| McFall et all (2017) | 14.0 | 7.6 | 20.4 |
| Panda et al (2014) | 42.0 | 37.6 | 46.4 |
| Sarna et al (2013) | 17.5 | 14.6 | 20.3 |
| **Heroin use** | | | |
| Kumar et al (2018) | 37.9 | 36.8 | 39.0 |
| McFall et all (2017) | 27.4 | 21.5 | 33.3 |
| Solomon et al (2008) | 84.5 | 81.9 | 87.1 |
| Mehta et al (2013) | 71.3 | 67.6 | 75.0 |
| **Having multiple sex partners** | | | |
| Ganesh et al (2020) | 6.9 | 2.1 | 11.6 |
| Kumar et al (2018) | 18.9 | 17.6 | 20.2 |
| McFall et all (2017) | 48.6 | 43.6 | 53.6 |
| Armstrong et al (2014) | 14.7 | 8.4 | 21.0 |
| Panda et al (2005) | 31.0 | 20.2 | 41.8 |
| Mehta et al (2013) | 32.6 | 26.9 | 38.3 |
| **Inconsistent condom use/ unsafe sexual practice** | | | |
| Ganesh et al (2020) | 11.4 | 6.8 | 16.0 |
| Kumar et al (2018) | 22.2 | 21.0 | 23.4 |
| Armstrong et al (2014) | 29.8 | 24.1 | 35.5 |
| Panda et al (2005) | 33.0 | 22.3 | 43.7 |

Note: 95%CI was obtained using the formula below.
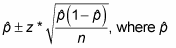

Supplement: Supplementary file 2 — Additional file 2. [file 12889_2022_13922_MOESM2_ESM.docx]
